# Sex-specific effects of reproductive season on bobcat space use, movement, and resource selection in the Appalachian Mountains of Virginia

**DOI:** 10.1371/journal.pone.0225355

**Published:** 2020-08-04

**Authors:** David C. McNitt, Robert S. Alonso, Michael J. Cherry, Michael L. Fies, Marcella J. Kelly

**Affiliations:** 1 Department of Fish and Wildlife Conservation, Virginia Tech, Blacksburg, Virginia, United State of America; 2 Virginia Department of Game and Inland Fisheries, Verona, Virginia, United States of America; US Geological Survey, UNITED STATES

## Abstract

Across taxa, sex-specific demands vary temporally in accordance with reproductive investments. In solitary carnivores, females must provision and protect young independently while meeting increased energetic demands. Males seek to monopolize access to females by maintaining large territories and defending them from other males. For many species, it is poorly understood how these demands relate to broad-scale animal movements. To investigate predictions surrounding the reproductive strategies of solitary carnivores and effects of local conditions on bobcat (*Lynx rufus*) spatial ecology, we examined the effects of sex and reproductive season on home range size, movement rate, and resource selection of bobcats in the central Appalachian Mountains. Male seasonal home ranges were approximately 3 times larger than those of females (33.9 ± 2.6 vs. 12.1 ± 2.4 km^2^, x±SE), and male movement rates were 1.4 times greater than females (212.6 ± 3.6 vs. 155 ± 8.2 m/hr), likely reflecting male efforts to maximize access to females. Both sexes appear to maintain relatively stable seasonal home ranges despite temporally varying reproductive investments, instead adjusting movements within home ranges. Males increased movements during the dispersal period, potentially reflecting increased territoriality prior to breeding. Females increased movements during the kitten-rearing period, when foraging more intensively, and frequently returning to den sites. Both sexes selected home ranges at higher elevations. However, females selected deciduous forest and avoided fields, whereas males selected fields and avoided deciduous forest, perhaps explained by male pressure to access multiple females across several mountain ridges and higher risk tolerance. Seasonal changes in home range selection likely reflect changes in home range shape. Increased female avoidance of fields during kitten rearing may indicate female avoidance of presumably resource rich, yet risky, fields at the time when kittens are most vulnerable. Our results indicate that while reproductive chronology influences the spatial ecology of solitary carnivores, effects may be constrained by territoriality.

## Introduction

An animal’s use of a landscape represents a series of life history tradeoffs, in which energy expenditure and mortality risk reduce fitness, and energy acquisition and reproductive success increase fitness [1]. Temporal variations in these tradeoffs correspond to varying demands required for reproductive success through time. For solitary carnivores, life history tradeoffs also vary widely between sexes due to differing reproductive investments [2]. Males must maximize access to females, and females must protect and provision young independently [2]. Both sexes must acquire sufficient energy to successfully execute these reproductive strategies. The implied costs of energy acquisition for carnivores are high, due to the need to hunt and kill prey [3,4]. The costs for females are highest during pregnancy and lactation, as these processes are energetically expensive [5]. Males sustain the highest costs during the breeding season when they must locate and breed females while defending territories against male competitors, in addition to foraging [2]. Considering these factors, the spatial ecology of male solitary carnivores is expected to be driven by the distribution of females and energy acquisition needs, whereas energy acquisition and protection of young should be the primary drivers of female spatial ecology. In the presence of a distinct breeding season, male spatial ecology should shift at that time to exploit the focal resource of receptive females. However, competition over females may continue year-round for territorial species, since the cost of maintaining an exclusive area could be lower than establishing one every breeding season [2]. Understanding how the reproductive chronology of solitary carnivores influences their spatial ecology can provide insight into fundamental ecological processes, since space use and resource selection can influence processes such as population dynamics, behavioral interactions, and foraging behavior [6–8].

Bobcats (*Lynx rufus*) are a solitary, midsized felid with a territorial social organization and polygynous breeding strategy [9]. Male bobcats exhibit larger home ranges than those of females [10], and may exhibit greater movement rates than females [11–14]. As obligate carnivores, bobcats require sufficient prey to meet energetic demands. Bobcats are known to select areas of dense vegetation that likely have higher prey availability [15–19]. Prey availability is considered the primary driver of regional variation in bobcat home range size; specifically, as prey availability increases, individuals can meet energetic demands in smaller areas [10,13,15].

Bobcat space use may vary seasonally, with seasonal variation being more common in northern latitudes with greater winter severity [19–22]. When home range sizes vary seasonally, they are typically smaller during summer months when prey is more available and females remain closer to den sites, and larger during winter months when prey may be less available and males seek to maximize breeding opportunities [14,19,22]. Although females may have smaller home ranges during the kitten-rearing season, their activity and movement rates often increase during this time, indicating more intensive use of home ranges [23–25]. Both sexes may increase movement rates during winter months, which has been attributed to breeding behavior and decreased prey availability [26,27]. Resource selection patterns often shift seasonally as a result of changes in the distribution and abundance of prey [11,17,19,22].

Bobcat populations are increasing or stable throughout much of their range [28]. These trends are evident in the central Appalachian Mountains [28]. Although bobcats largely persisted, wolves (*Canis* spp.) and cougars (*Puma concolor*) remain extirpated from ecosystems in the Appalachian Mountains, leaving bobcats as an apex predator in the region, along with black bears (*Ursus americanus*) and coyotes (*Canis latrans*) [29]. Within this guild, bobcats are the only obligate carnivore, can occur at population densities approximately twice those of coyotes [30,31], and have the broadest dietary niche of prey items [29]. Thus, bobcats hold potential to influence processes relating to both prey and competitors, and understanding the spatial ecology of bobcats may be important to predicting spatial variation in food web dynamics in these systems. In addition to their ecological importance, bobcats have economic and socio-cultural value as a furbearer and game species in the central Appalachian Mountains and are generally valued by the public [32]. Despite their importance, there is a paucity of information on bobcat spatial ecology throughout the central Appalachian Mountains.

We examined the sex- and season-specific aspects of bobcat spatial ecology in western Virginia to gain insight into the life history tradeoffs of solitary carnivores, how these tradeoffs may vary from other regions, and what conditions drive those variations. We estimated home ranges to quantify the space required to acquire resources, calculated movement rates to examine the intensity of home range use, and conducted resource selection analyses to determine the drivers of home range selection (i.e. 2^nd^ order resource selection [8]). Males should seek to monopolize access to multiple females, thus we expected male home ranges to be larger than those of females, male movement rates to be greater than those of females, and both metrics to increase during the breeding season. Since females face increased energetic demands during kitten rearing, but also must balance provisioning and protecting young, we expected female home range size to decrease and movement rates to increase during kitten rearing. Female home range selection should be driven by prey and protection of young, whereas male home range selection should be driven by access to females and prey, therefore resource selection patterns should differ between sexes. Seasonal resource selection should be driven by shifting prey use and availability for both sexes, but may also be influenced by female protection of young and male breeding behavior.

## Materials and methods

### Study area

**Fig 1:**
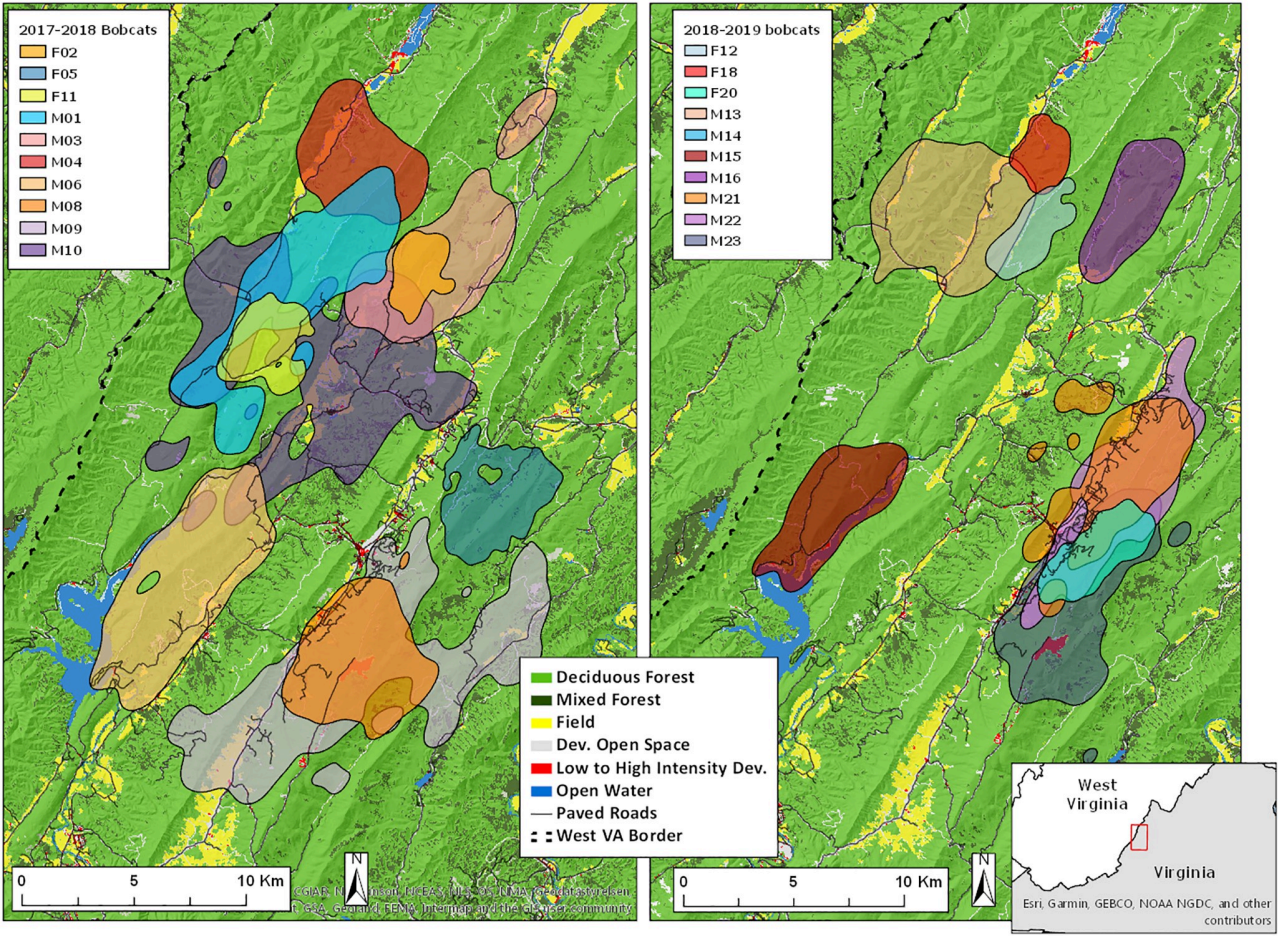
Map of study area with land cover and 95% home ranges of bobcats (n = 20) monitored from 2017–2019 in Bath County, VA. Home ranges were calculated using the autocorrelated kernel density estimator.

Our study area encompasses the western half of Bath County, Virginia, adjacent to the border with West Virginia (Fig 1). Bath County is in the Valley and Ridge physiographic province of the Appalachian Mountain range, characterized by parallel, northeast-southwest oriented ridges with narrow valleys interspersed. The repetitive topographical pattern results in largely predictable land cover, with public, forested land on the steep ridges and slopes, and narrow strips of private, low-intensity development and agriculture in the flatter valley bottoms. Bath County is 90% forested land cover, most of which is managed by the US Forest Service and Virginia Department of Game and Inland Fisheries. Elevation ranges from 343 meters to 1363 meters. Average monthly temperature can range from -1 to 22 °C, with a mean daily minimum temperature of -7 °C in January and a mean maximum temperature of 28 °C in July [33]. Average annual precipitation is 110 cm [33]. Forest structure primarily consists of mature deciduous forest, with common overstory species including oak (*Quercus* spp.), hickory (*Carya* spp.), maple (*Acer* spp.), and tulip poplar (*Liriodendron tulipifera*). Conifers are present in some forest stands, with common species including pine (*Pinus* spp.) and hemlock (*Tsuga* spp.). Other than bobcats, the large carnivore guild includes coyotes and black bears. Common bobcat diet items are squirrels (*Sciurus* spp.), voles *(Microtus* spp., *Myodes gapperi)*, mice (*Peromyscus* spp.), cottontail rabbits *(Sylvilagus* spp.), and white-tailed deer (*Odocoileus virginianus*) [29].

### Bobcat capture and monitoring

We captured bobcats using cage traps (Camtrip Cages, Bartsow, California, USA and Briarpatch Cages, Rigby, Idaho, USA) in accordance with Virginia Tech IACUC protocol #16–071. Capture was conducted during 2017 and 2018, primarily during late winter and spring, with a small number of captures during autumn. We checked traps twice daily (early morning and evening). We immobilized bobcats with a mixture of 10–15 mg/kg ketamine hydrochloride and 1mg/kg xylazine using hand injection with a syringe. We monitored and recorded respiratory rates, heart rates, and temperatures every 5–10 minutes. We used tooth growth and condition, body morphology, and teat/scrotum characteristics to determine whether bobcats were juvenile or adult [34]. We fitted adult bobcats with Iridium GPS collars (Telonics, Mesa, Arizona, USA and Advanced Telemetry Systems, Isanti, Minnesota, USA). All bobcats captured were marked with color-coded numbered ear tags. Following handling, we reversed xylazine with 0.125 mg/kg yohimbine, administered either rectally or intramuscularly, and allowed bobcats to recover in the cage trap for 30 minutes to 1 hour before release. We programmed GPS collars to collect locations at 1, 2, and 4-hour intervals, however finer-scale sampling was related to other research objectives. For these analyses we used standardized 4-hour intervals.

### Reproductive season classification

We classified 3 seasons of interest (breeding, kitten-rearing, and dispersal) based on the reproductive chronology and life history of bobcats. We classified January 1—March 31 as the breeding season, to overlap with the estrus cycle of females. Bobcats are spontaneous ovulators, with the peak of estrous occurring in February and March [35]. To maximize reproductive success, males must breed as many females as possible during this window. Once pregnant, energetic demands will begin to increase for females [5]. We classified kitten-rearing season as April 1—September 30, since the 60–70 day gestation period results in parturition during April and May. Kittens will feed exclusively on milk for approximately their first 2 months, then they will nurse daily and consume small prey delivered by the mother for an additional 2 months, learning to hunt in the later phases of this period [36]. It is evident that kittens rely heavily on their mother during this approximately 4-month period, and that kitten-bearing females are under significant pressure to acquire abundant prey. We classified the dispersal season as October 1—December 31. Similar to the approach of Chamberlain et al. [11], we examined this third season (what they termed “winter”) in addition to breeding and kitten-rearing. Presumably, resident females seek to restore body mass depleted during the kitten-rearing period, and resident males will aim to maximize body mass in preparation for the breeding season. Although dates of dispersal initiation can vary widely [37–40], pressure for dispersers to establish home ranges should be highest prior to the breeding season, since natal dispersal is defined by movements to reproductive sites [41]. Thus, the months of October through December represent a distinct period of bobcat behavior.

### Home range analysis

We estimated bobcat home ranges using the autocorrelated kernel density estimator (AKDE) [42] using the continuous-time movement modeling package (ctmm) [43] in program R version 3.5.3 [44]. The AKDE is a third-generation estimator that assumes the data represent a sample from a nonstationary, autocorrelated continuous movement process by incorporating the movement of animals through an autocorrelation function derived from movement models fit to the data [42]. Furthermore, AKDE reduces to a conventional kernel density estimator when locations are truly independent, and can correct for missing locations and irregular sampling schedules through an optimal weighting method [45]. We estimated 95% annual home ranges for bobcats with at least 4 months of relocation data, during at least 2 seasons. We estimated 95% seasonal home ranges for bobcats with locations collected for at least 1 month in a given season. The autocorrelation structure of the data indicates that approximately 2 weeks of movement data is sufficient to estimate bobcat home ranges, supporting these thresholds (S1 Fig in S1 Appendix). We fit linear mixed effects models for each season using restricted maximum likelihood, with area of 95% seasonal home range as the response variables. We used a natural logarithm transformation for home range sizes to meet assumptions of normality. We included both the interaction and main effects of sex and season as predictors, and treated animal-specific intercepts as random effects. We fit models in the program R [44] package lme4 [46] and assessed the significance of factors and degrees of freedom using Satterthwaite’s method for approximating degrees of freedom in the program R [44] package lmerTest [47].

### Movement analysis

We estimated each bobcat’s movement rates in meters moved per hour, calculated as the straight-line step length between successive locations divided by the time lag. Only steps with a 4-hour time lag were included. We calculated annual movement rates to facilitate comparisons to other studies and we calculated seasonal movement rates to examine seasonal effects. We calculated annual movement rates for bobcats with at least 4 months of relocation data during at least 2 seasons. Seasonal movement rates were only examined for bobcats that were monitored for at least one month in a given season. We assigned each step to the appropriate season. We used a generalized linear mixed effects model (GLMM) in the program R [44] package lme4 [47], with a gamma distribution and log link to model movement rates as a function of the direct and interactive effects of sex and season. We treated animal-specific intercepts as random effects. Significance of covariates was determined using Wald Z-test.

### Resource selection analysis

**Fig 2:**
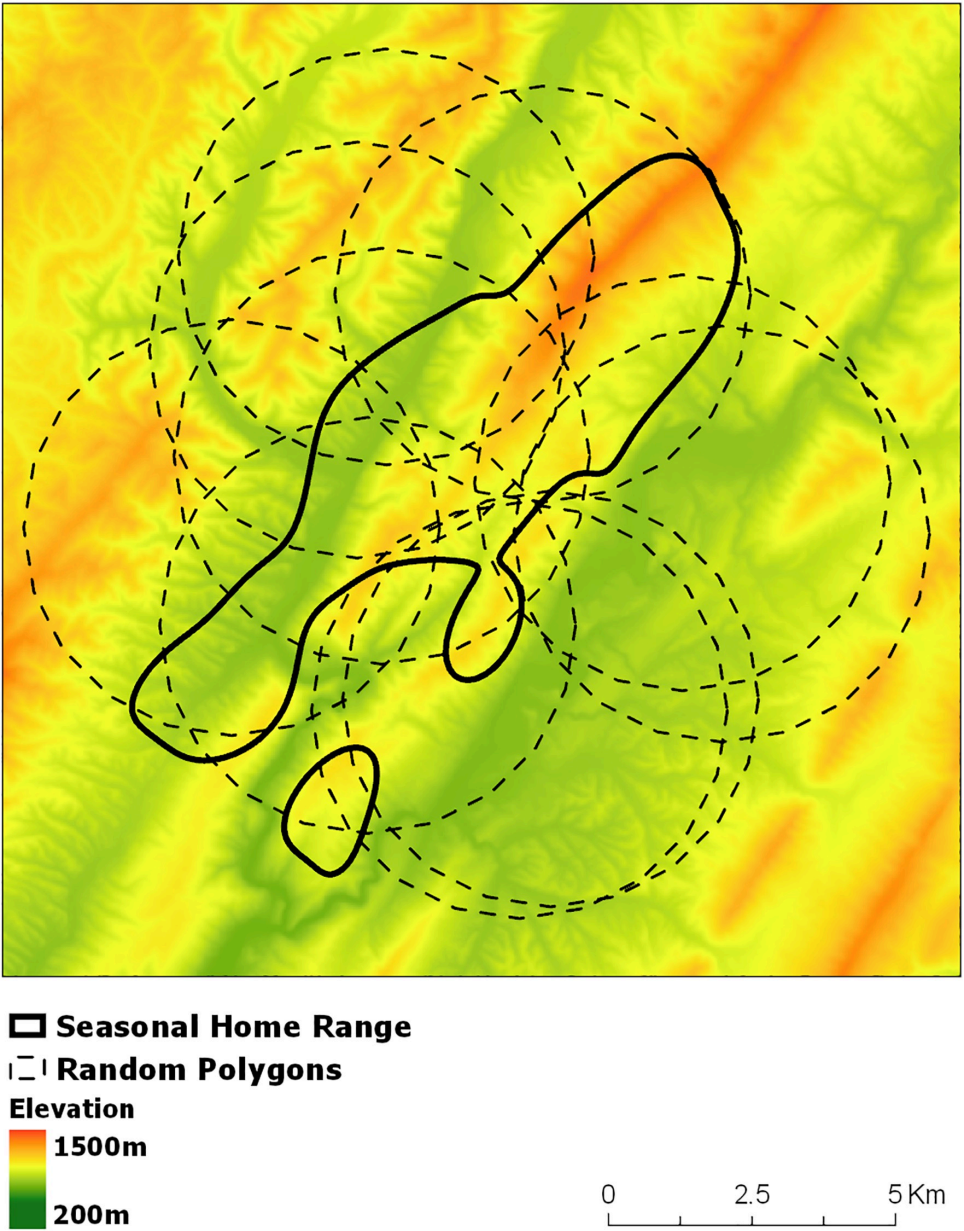
Example of method used to estimate use and availability for 2^nd^ order resource selection functions. Polygons were randomly simulated within a constrained area (5.3km) surrounding seasonal home ranges. Constraining distance was based on observed space use of 2 dispersing bobcats. Covariates were sampled within actual home ranges and within simulated polygons and compared. Base layer consists of a 30 m DEM to show elevation as an example covariate.

We examined seasonal bobcat selection of home ranges within the landscape (2^nd^ order [8]) by creating resource selection functions (RSF) in a use-availability framework [48]. We characterized resource availability by simulating random circular polygons, following Katnik and Wielgus’ [49] assertion that randomly located, simulated home ranges estimate availability more accurately than landscape proportions. To define availability for each bobcat in each season we simulated 10 polygons equivalent in size to each animal’s seasonal home range. We constrained the available polygon centers within a 5.3 km distance from each seasonal home range center (Fig 2). We based the constraining distance of 5.3 km on the radius of the largest home range (88.5 km^2^) of 2 dispersing bobcats that we collared. We classified the 2 male bobcats as dispersers due to prolonged erratic movements that resulted in home ranges more than twice as large as the male average. This area should reflect available habitat more accurately than using the entire study area, as we did not randomly or systematically sample the study area due to trapping access. Our approach is not susceptible to bias associated with the distribution of our trapping effort, since availability is individual specific (i.e. available home ranges were constrained to be proximate to a given animal’s observed seasonal home range) and the proximity constraint was based on a case study of two bobcats dispersing (i.e., selecting a home range) in our study area. The time between dispersal initiation and settlement is a crucial aspect of home range selection [50]. Following the simulation of polygons, we systematically extracted covariate values from every 10^th^ raster cell within both simulated and real seasonal home ranges. In our study area, there are no systematic landscape patterns (e.g. road grids) at this scale, and when a sample of all cells is compared to a systematic sample of every tenth cell the values of a given landscape attribute are nearly identical [S1 Table in S1 Appendix].

#### Resource selection data

We included land cover and topographical based covariates in the resource selection functions. Land cover covariates included were distance to deciduous forest, distance to mixed forest, and distance to fields, which we derived from the 30 m resolution 2011 National Land Cover Database (NLCD). These cover types compose the vast majority of the study area. Previous research in the eastern United States suggests that bobcats select for forest habitat [11,12,51,52]. We decided to delineate between mixed and deciduous forest because evergreen vegetation can be a primary source of concealment cover when deciduous woody vegetation and herbaceous groundcover is limited during the dormant season. Areas without forest cover typically consist of fields, which are mostly cattle pastures and hay fields. The type of dense vegetation that bobcats often select for is rare within these open fields, but field edges may provide dense vegetation. Row crops were effectively absent from the study area. We did not include development since development only composes approximately 1% of the study area and is mostly clustered in small, localized areas. The deciduous forest covariate consisted of the Deciduous Forest class in the NLCD. To create a mixed forest covariate, we combined the Evergreen Forest and Mixed Forest NLCD classes. To create the field covariate, we combined the Pasture/Hay and Cultivated Crops NLCD classes. Lastly, we created distance raster layers by calculating Euclidean distance to each land cover type using the Euclidean Distance tool in ArcGIS 10.6 (ESRI, Redlands, CA, USA). We used distance-based land cover covariates because they remove the need to base inference on reference categories, reduce the influence of telemetry error, and because effects of land cover types can extend beyond their boundaries (e.g. edge effects surrounding fields) [53]. Topographical covariates included elevation and slope at a 30 m resolution [54]. Private lands were almost exclusively at lower elevations and public lands at higher elevations, thus elevation can also offer insight into the influence of land use in the consistent land cover pattern of the Valley and Ridge province. Slope provides insight into the influence of mountainous terrain on bobcat resource selection, due to implicit costs of movement on steeper slopes. Steep slopes also can influence movement by providing concealment and limiting accessibility to humans and other predators. We extracted elevation values from a digital elevation model at a 30 m resolution (DEM, United States Geological Survey 2013). We calculated slope using the DEM with the Slope tool in ArcGIS 10.6 (ESRI, Redlands, CA, USA), which resulted in a 30 m resolution.

#### Resource selection model development

To examine 2^nd^ order bobcat resource selection, we developed RSFs using binomial generalized linear mixed models (GLMM) in Program R [44] package lme4 [46]. The binary response variable for resource selection was whether a raster cell was extracted from an observed seasonal home range (used = 1) or a simulated polygon (available = 0). Predictor variables were distance to deciduous forest, distance to mixed forest, distance to fields, elevation, and slope. No covariates were highly correlated (all r < ǀ0.5ǀ; Pearson’s correlation). We created separate models for male and female bobcats, each consisting of the 5 main effects, and tested if reproductive season influenced selection by including season as a main effect and interaction term.

We rescaled all covariates by mean-centering at zero then dividing them by their standard deviation to facilitate model convergence. We included animal-specific random intercepts to account for variation in sampling duration among individuals [55]. We evaluated selection or avoidance based on whether a coefficient significantly differed from zero (α = 0.05). We determined significance of covariates using Wald Z-test. We inferred selection if used points were closer to habitat variables than expected, and avoidance if used points were further from habitat variables than expected. We compared coefficient estimates within models, from largest to smallest, to evaluate relative importance of the various covariates.

## Results

We deployed GPS collars on 20 bobcats (14 male, 6 female) from January 2017 through April 2018. Number of locations per bobcat ranged from 259 to 1979, with a mean of 933. Length of collar deployments ranged from 55–393 days, with a mean deployment length of 259 days.

**Fig 3:**
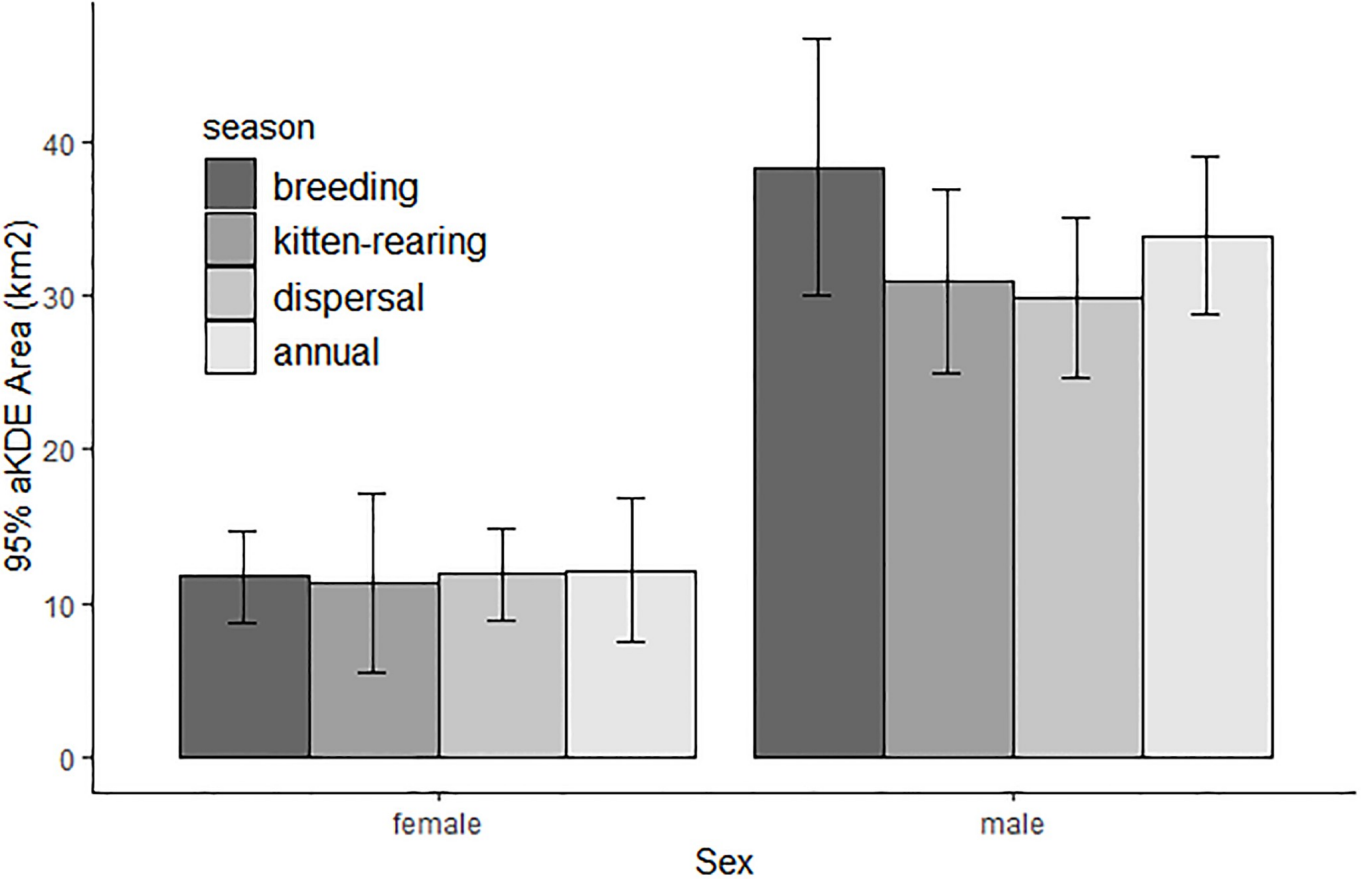
Means and 95% confidence intervals of 95% home ranges of female and male resident bobcats monitored during 2017–2019 in Bath County, VA, for breeding (n = 12, 4 females, 8 males), kitten-rearing (n = 16, 5 females, 11 males), and dispersal/pre-breeding (n = 15, 4 females, 11 males) seasons, and annual (n = 16, 5 females, 11 males). Home ranges calculated using the autocorrelated kernel density estimator.

We estimated home ranges for 16 resident bobcats (11 males, 5 females) and 2 dispersing males, excluding 2 bobcats (1 male, 1 female) that were monitored for less than 4 months and only during 1 season. We estimated 41 seasonal home ranges, including 13 bobcats in the breeding season (8 males, 5 females), 15 bobcats in the kitten-rearing season (11 males, 4 females), and 13 bobcats in the dispersal season (9 males, 4 females). The minimum number of locations used to calculate a seasonal home range was 129. On average, resident male home ranges were 33.9 ± 2.6 km^2^ (x ± SE) and were approximately 3 times larger than resident female home ranges (12.1 ± 2.4 km^2^, Fig 3). The home ranges of the 2 dispersing males were 84.8 and 88.5 km^2^ and both individuals exhibited prolonged, erratic movements on the landscape. Male home ranges were larger than female home ranges during all seasons and there was no significant effect of season on home range size, although male home ranges were slightly larger during the breeding season (Table 1, Fig 3).

**Table 1:** Linear mixed model for bobcats monitored during 2017–2019 in Bath County, VA with log transformed home range area as response and reproductive season interacting with sex as predictors. Reference categories are sex = female and season = dispersal.

Covariate	Β	SE	df	t value	Pr(>|t|)
Intercept	2.370	0.202	26.95	11.755	< 0.001
Breeding Season	0.020	0.177	21.62	0.111	0.913
Kitten-rearing season	-0.020	0.190	24.51	-0.103	0.918
Male	1.098	0.236	26.69	4.658	< 0.001
Breeding season x male	0.231	0.215	22.04	1.073	0.295
Kitten-rearing season x male	0.064	0.217	23.99	0.295	0.770

**Fig 4:**
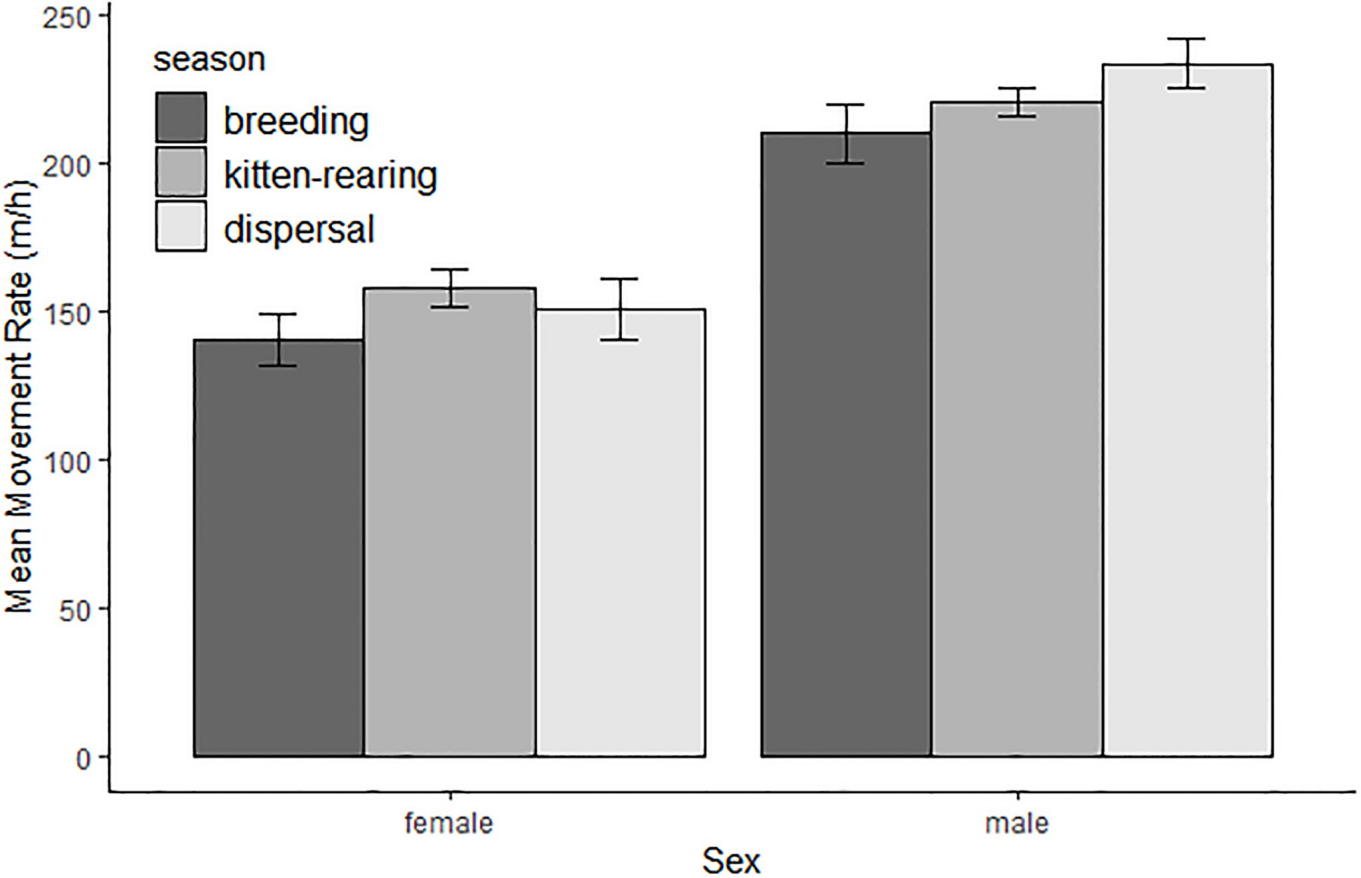
Means and 95% confidence intervals for movement rates of female and male bobcats monitored during 2017–2019 in Bath County, VA, for breeding (n = 14, 5 females, 9 males), kitten-rearing (n = 17, 4 females, 13 males), and dispersal/pre-breeding (n = 15, 4 females, 11 males) seasons. Movement rate is reported as meters moved per hour (m/h).

We estimated annual movement rates for 18 bobcats (13 males, 5 females), and seasonal movement rates for bobcats with at least 1 month of relocations within a given season. Mean male movement rates (212.6 ± 3.6 meters/hour) were approximately 1.4 times greater than mean female movement rates (155.4 ± 8.2 meters/hour). Male movement rates were higher than female movement rates during all seasons, and female movement rates were significantly higher during the kitten-rearing season while male movement rates were significantly higher during the dispersal season (Table 2, Fig 4).

**Table 2:** Gamma generalized linear mixed-effects model for bobcats monitored during 2017–2019 in Bath County, VA, with movement rates as response and reproductive season interacting with sex as predictors. Reference categories are sex = female and season = kitten-rearing.

Covariate	β	SE	t value	Pr(>|z|)
Intercept	5.127	0.099	51.867	< 0.001
Breeding season	-0.150	0.072	-2.093	0.036
Dispersal season	-0.149	0.057	-2.605	0.009
Male	0.294	0.116	2.535	0.011
Breeding season x male	0.082	0.078	1.060	0.289
Dispersal season x male	0.189	0.064	2.968	0.003

**Fig 5:**
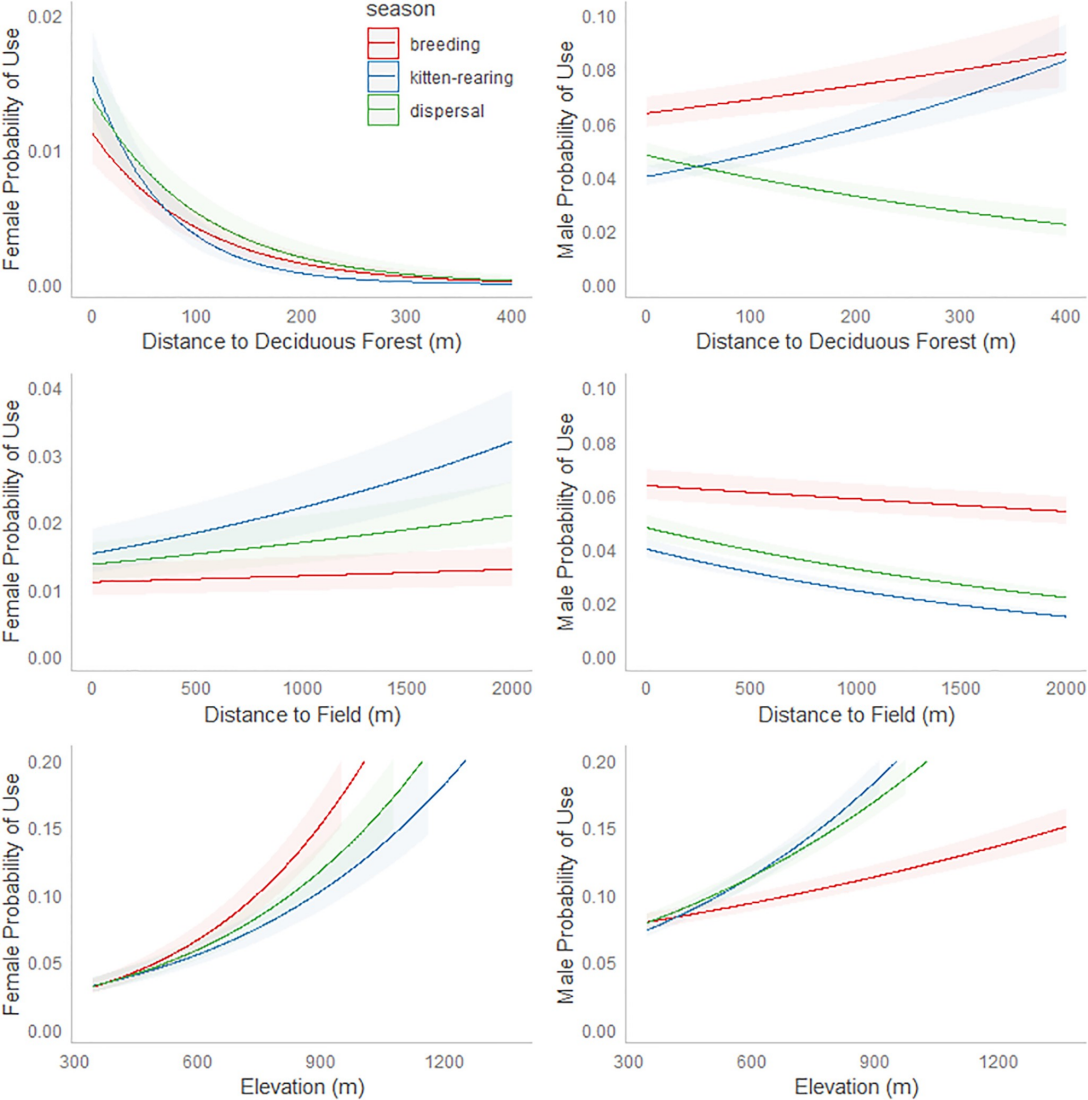
Relative probability of 2^nd^ order selection with 95% confidence intervals for female bobcats monitored during 2017–2019 in Bath County, VA, for breeding (5 females, 9 males), kitten-rearing (4 females, 13 males), and dispersal/pre-breeding (4 females, 11 males) seasons. The three strongest covariates are included (elevation, distance to deciduous forest, distance to fields). See Table 3 for all covariates.

We examined selection of seasonal home ranges for all resident bobcats (12 males, 6 females), which excluded 2 dispersing males. For females, distance to deciduous forest, distance to fields, and elevation were the strongest predictors of home range selection (Table 3, Fig 5. During all seasons, females selected home ranges that were at higher elevations, closer to deciduous forest, and farther from fields than expected (Table 3, Fig 5). Females exhibited strongest selection for deciduous forest during the kitten-rearing season (Table 3, Fig 5). Females exhibited strongest avoidance of fields during the kitten-rearing season, weaker avoidance of fields during the dispersal season, and weakest avoidance of fields during the breeding season (Table 3, Fig 5). Females exhibited strongest selection for higher elevations during the breeding season, less strong selection for high elevations during the dispersal season, and weakest selection for high elevations during the kitten-rearing season (Table 3, Fig 5). Females exhibited strongest selection for mixed forest during the dispersal and breeding seasons, but did not select or avoid mixed forest during the kitten-rearing season (Table 3). Females exhibited selection for steeper slopes during the dispersal season, but exhibited selection for gentler slopes during the breeding and kitten-rearing seasons (Table 3).

**Table 3:** Model results for 2^nd^ order resource selection functions (RSF) for 18 bobcats (12 male, 6 female) collared in Bath County, Virginia in years 2017–2019, including separate models for males and females. RSF models are binomial generalized linear mixed-effects models. Results include β coefficients (β), and standard errors (SE), z values, and p values from Wald tests. Reference category is season = dispersal.

Sex	Covariate	β	SE	Z value	P value
	Intercept	-2.346	0.072	-32.546	< 0.001
*Female*	Deciduous	-0.244	0.027	-8.998	< 0.001
	Mixed	-0.124	0.015	-8.553	< 0.001
	Field	0.166	0.014	11.900	< 0.001
	Elevation	0.381	0.016	24.386	< 0.001
	Slope	0.044	0.016	2.796	0.005
	Breed	0.023	0.022	1.027	0.304
	Deciduous x breed	-0.006	0.037	-0.154	0.878
	Mixed x breed	0.052	0.020	2.674	0.008
	Field x breed	-0.105	0.020	-5.162	< 0.001
	Elevation x breed	0.086	0.021	4.086	< 0.001
	Slope x breed	-0.053	0.023	-2.341	0.019
	Kitten	-0.071	0.026	-2.764	0.006
	Deciduous x kitten	-0.126	0.043	-2.943	0.003
	Mixed x kitten	0.137	0.024	5.679	< 0.001
	Field x kitten	0.124	0.021	5.781	< 0.001
	Elevation x kitten	-0.045	0.022	-2.064	0.039
	Slope x kitten	-0.116	0.023	-5.043	< 0.001
	Intercept	-2.325	0.037	-62.647	< 0.001
*Male*	Deciduous	-0.054	0.008	-6.928	< 0.001
	Mixed	-0.065	0.007	-9.933	< 0.001
	Field	-0.293	0.008	-38.014	< 0.001
	Elevation	0.234	0.006	36.515	< 0.001
	Slope	-0.003	0.007	-0.490	0.624
	Breed	0.014	0.009	1.529	0.126
	Deciduous x breed	0.076	0.010	7.864	< 0.001
	Mixed x breed	0.002	0.009	0.268	0.789
	Field x breed	0.229	0.010	23.579	< 0.001
	Elevation x breed	-0.128	0.009	-14.721	< 0.001
	Slope x breed	0.010	0.009	1.108	0.268
	Kitten	-0.011	0.009	-1.285	0.199
	Deciduous x kitten	0.107	0.009	11.349	< 0.001
	Mixed x kitten	0.025	0.009	2.878	0.004
	Field x kitten	-0.075	0.010	-7.492	< 0.001
	Elevation x kitten	0.048	0.008	5.767	< 0.001
	Slope x kitten	-0.011	0.009	-1.196	0.232

For males, distance to fields and elevation were the strongest predictors of home range selection (Table 3, Fig 5). During all seasons, males selected home ranges that were closer to fields and at higher elevations than expected (Table 3, Fig 5). Males exhibited strongest selection for fields during the kitten-rearing season and weakest selection for fields during the breeding season (Table 3, Fig 5). Males exhibited weakest selection for high elevations during the breeding season compared to dispersal and kitten-rearing seasons (Table 3, Fig 5). Males exhibited selection for mixed forest during all seasons, but this selection was weakest during the kitten-rearing season, following a similar pattern as females (Table 3). Males exhibited selection for deciduous forest during the dispersal season, but avoided deciduous forest during breeding and kitten-rearing seasons (Table 3, Fig 5). Slope was not a significant predictor of male home range selection (Table 3).

## Discussion

As predicted, male space use requirements were greater than those of females during all seasons. However, reproductive season did not have a significant effect on home range size for either sex, contrary to our predictions. Instead, both sexes appeared to use home ranges more intensively during different reproductive seasons, specifically the kitten-rearing season for females and the dispersal season for males. The factors influencing selection of home ranges on the landscape did differ between sexes, as predicted. Home range selection patterns also varied among seasons, and these seasonal variations were sometimes opposite for each sex, indicating that they are driven by factors beyond shifting prey availability.

To maximize reproductive success, bobcats must acquire energy while mitigating risk. Increased food intake, and resulting increases in body mass and nutritional reserves, may increase reproductive success through outcompeting conspecifics of the same sex, increased litter size, and increased body mass of neonates [56]. Male bobcats must maintain sufficient body mass and locomotive energy to monopolize access to multiple females. Female bobcats require consistent energy acquisition for successful pregnancy and kitten rearing, while minimizing risk to kittens. Male bobcats in our study had larger home ranges than females, presumably to maximize breeding opportunities with females. Although male home ranges were slightly larger on average during the breeding season, their home range size did not differ significantly between breeding and non-breeding seasons. This lack of seasonal variation supports the prediction that it is less costly to maintain large territories year-round than to reestablish them each year prior to the breeding season. If males increased territories immediately prior to the breeding season, it is more likely they would be nutritionally drained or wounded by conspecifics as they enter the brief window of estrus. Likewise, female home range size remained static across seasons, even though energetic demands increase during kitten-rearing. Despite the stability in size, there were some seasonal variations in home range shape, evidenced by seasonally shifting resource selection patterns. Perhaps it is too costly to shift territories, but bobcats will alter use of the broader home range, making close forays to capitalize on prey or receptive females. Seasonal changes in home range size or location are typically found in far northern latitudes [13,19,21,22], where harsh winters may severely limit prey availability. Conner et al. [57] suggested that when prey falls below a “critical availability”, emigration or mortality must occur. Our findings likely reflect prey availability above this threshold for the duration of our study, and reinforces that home range stability is more tenuous towards the northern limits of the bobcat distribution.

Bobcats in our study area used home ranges more intensively during certain periods, indicated by seasonal increases in movement rates for both sexes. The increased female movement rates we observed during the kitten-rearing season are likely due to the need to increase foraging but also attend to young, resulting in frequent movements between dens and foraging sites. The energetic costs of lactation are extremely high [5], and female bobcat movements have been found to increase during kitten rearing [23]. Increased movement rates for resident males during the dispersal season may be due to increased territorial marking and patrolling in response to dispersing males. Physical territorial conflicts between bobcats are thought to be largely avoided by communicating through urine spraying, feces deposition, physical scrapes, olfactory investigation, and vocalizations [58].

Male movement rates were greater than female movement rates throughout the year. Locomotion is energetically costly, particularly for larger mammals traveling uphill [59]. Previous bobcat research conducted with temporally-coarse sampling rates often found greater male movement rates [13,14], but finer-scale telemetry has found mixed results. In low-relief areas of Mexico and North Carolina, there was a lack of sex effect on movement rates [24,60]. Male movement rates were greater in our mountainous study area, in the mountains of Vermont [12], and an area of Mississippi with up to 20% slope [11]. Sex effects on movement rates may be more pronounced in rugged topography, as females further seek to reduce energetic costs associated with locomotion, whereas males still seek to maximize access to multiple females by maintaining large territories. This aligns with Sikes and Kennedy [61] findings that eastern bobcats are more sexually dimorphic in size in mountainous areas, and their suggestion that this is caused by selective pressure for smaller female body size to minimize increased energetic costs of locomotion in rugged terrain. On average, males had 1.5 times greater body mass than females in our study [62]. Further, we found females avoided steep slopes during the breeding and kitten-rearing seasons, but we did not observe this pattern in males. More data is needed to further elucidate these patterns and their driving factors. Many studies of bobcat space use have not reported sex-specific movement rates.

Both male and female bobcats strongly selected home ranges at higher elevations. In the Valley and Ridge province of the Appalachian Mountains higher elevation ridges are almost entirely public, undeveloped land while valleys are typically private and consist of agriculture and development. Conversion of valley bottoms from rich riparian forests to agriculture and development has probably shifted bobcat space use from historical patterns, leading to increased use of ridges. In addition to decreased habitat quality, risk may be higher in valley bottoms due to human land use patterns. Legal harvest and vehicle collisions, the leading causes of mortality on our study, were most common in or near valleys.

Other resource selection patterns differed between sexes. Male bobcats exhibited selection for fields and avoidance of deciduous forest, whereas female bobcats contrastingly exhibited avoidance of fields and selection of deciduous forest. These opposing trends may be explained by the valley and ridge topography of the study area, combined with differing reproductive pressures and risk tolerances. Female home ranges almost exclusively occurred within a single ridge, whereas male home ranges often contained multiple ridges (Fig 1), perhaps reflecting attempts to overlap multiple female home ranges. In moving between ridges, male bobcats must cross valleys and therefore encompass fields within their home ranges. Chamberlain et al. [11] found male bobcat resource selection to vary by scale, and suggested that males may select home ranges primarily based on the spatial distribution of females, which aligns with the space use trends we observed. It is important to note that approximately 90% of the study area consists of forested land cover, and although male bobcats did not use it more than available at a landscape scale, forest was still heavily used.

Resource selection patterns also varied seasonally for both sexes, with opposing trends sometimes evident. Male selection for fields and avoidance of deciduous forest increased during summer (i.e. kitten rearing), whereas contrastingly, female avoidance of fields and selection for deciduous forest increased during kitten-rearing. The kitten-rearing season coincides with the presence of white-tailed deer fawns, small mammals, and juvenile rabbits, all of which are likely to be found in areas of dense vegetation along field edges [63–66]. Thus, males are likely closer to fields than expected during summer to exploit these pulsed food resources. The opposing trends we observed in females may represent female avoidance of male bobcats when kittens are most vulnerable, which is presumably the time surrounding nursing and during the first forays away from dens [67]. Although not yet observed in bobcats, infanticide has been observed in many felid species, including lynx (*Lynx* spp.) [68,69]. Additionally, black bears and coyotes are well-documented fawn predators [70–74], and eastern coyotes can be more abundant in open areas, including the types of pasture found in our study area [75]. Thus, sympatric carnivore species may be frequenting areas near fields at this time, leading to additional risk for female bobcats with kittens. Kitten survival is a crucial component of lifetime reproductive success, and female felids will adjust behavior preemptively to ensure survival of young [67,76–78].

Some seasonal resource selection patterns were the same for both sexes, and were likely influenced by shifts in the distribution of prey. Prey distribution is a main driver of bobcat resource selection [79]. Both sexes exhibited selection for mixed forest during the dispersal and breeding seasons, but they either exhibited weaker selection for, or did not select for, mixed forest during the kitten-rearing season. Other studies have found bobcat selection for conifers during winter months, which has been attributed to relatively higher concealment cover and prey availability during winter [22,80,81]. Both sexes also selected deciduous forest during the dispersal season, which overlaps with the peak of hard mast production. Squirrels are the most common diet item of bobcats in this study area [29], and both gray squirrels (*Sciurus carolinensis*) and fox squirrels (*Sciurus niger*) exhibit peaks in foraging behavior during this time [82–84].

By examining home ranges, movements, and resource selection across sex and reproductive season, we gained insight into the influence of life-history tradeoffs on bobcat spatial ecology. A limitation of our study was the relatively small sample size of individuals, particularly of female bobcats. Despite this, we observed sex and seasonal effects on space use, indicating that these effects are strong. Future research should further examine the causal factors of sex effects on movement rates and investigate possible avoidance of male bobcats by females when rearing kittens.

## Supporting information

S1 Appendix (DOCX)

S1 Data (CSV)
