# Supplementary material for: Sex-specific effects of reproductive season on bobcat space use, movement, and resource selection in the Appalachian Mountains of Virginia
Source: PLoS One. 2020 Aug 4;15(8):e0225355. doi: 10.1371/journal.pone.0225355 (PMC7402482; doi:10.1371/journal.pone.0225355)
Supplement: S1 Appendix — (DOCX) [file pone.0225355.s001.docx]

**Appendices**

S1 Fig. Population variogram for bobcats (6 female, 14 male). The variogram asymptotes at approximately 2 weeks, indicating multiple home range crossings by this time.

S1 Table. Comparison of covariates sampled from all raster cells versus every 10^th^ raster cell within polygons for landscape-scale (2^nd^ order) resource selection analysis. Included mean and median values for distance to deciduous forest, distance to mixed forest, distance to fields, elevation, and slope.

| Variable | All cells | Every 10th cell |
| --- | --- | --- |
| Deciduous distance mean | 8.22 | 8.21 |
| Mixed distance mean | 345.68 | 345.68 |
| Field distance mean | 930.85 | 930.88 |
| Elevation mean | 727.84 | 727.84 |
| Slope mean | 16.03 | 16.04 |
| Deciduous distance median | 0.00 | 0.00 |
| Mixed distance median | 271.66 | 271.66 |
| Field distance median | 787.46 | 787.46 |
| Elevation median | 710.91 | 710.88 |
| Slope median | 15.81 | 15.83 |
